# P-828. Financial and Environmental Impact of Duplicate Hepatitis C Antibody Tests

**DOI:** 10.1093/ofid/ofaf695.1036

**Published:** 2026-01-11

**Authors:** Soo Jin Moon, Pamela S Lee, Timothy Hatlen

**Affiliations:** Harbor-UCLA Medical Center, Torrance, CA; Harbor UCLA, Torrance, California; Harbor-UCLA Medical Center, Torrance, CA

## Abstract

**Background:**

There are limited data on the impact of duplicate Hepatitis C antibody (HCV Ab) testing in the era of universal HCV screening. We quantified unnecessary repeat HCV Ab tests and estimated the associated financial cost, healthcare waste, and carbon emissions.

**Figure:**
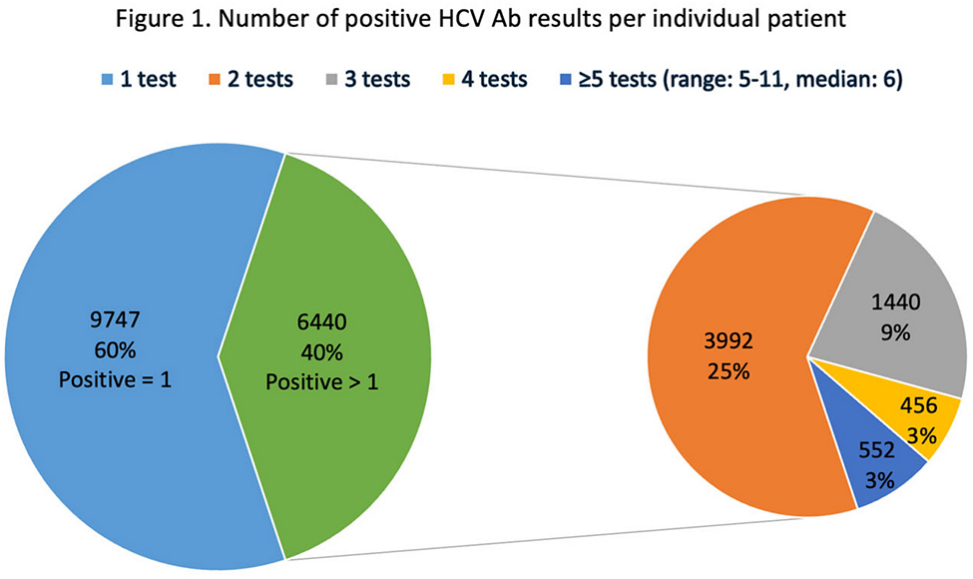

**Figure:**
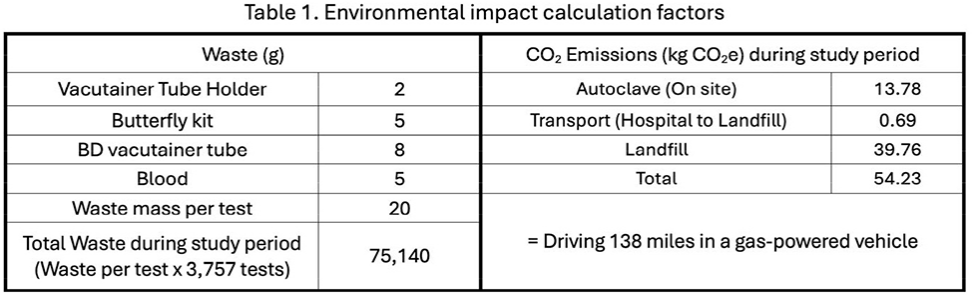

**Methods:**

We quantified positive HCV Ab test results for each patient from the Los Angeles County Department of Health Services (LAC DHS) system from 1/1/2018 to 6/30/2024. Unnecessary tests included those repeated with more than one positive result.

We identified and weighed all biohazardous materials from HCV Ab testing. We calculated greenhouse gas emissions assuming: 1) Materials were autoclaved (0.84 MWh/ton electricity) then transported to a landfill 50 miles away; 2) Electricity emission factor was 436.7 lbs/MWh. HCV Ab test cost was $5.05 (Quest Diagnostics).

**Results:**

A total of 355,060 HCV Ab tests were performed with 16,187 reactive, 338,161 nonreactive, and 614 equivocal results. Among the reactive results, 9,747 (60%) patients tested positive once, and 6,440 (40%) patients tested positive twice or more [Figure 1]. A total of 3,757 tests were performed unnecessarily.

Plastic waste from unnecessary tests was 20g per test, or 75.14kg of total waste. Emissions from the disposal of redundant tests caused 54.23 kg CO_2_ emissions, equivalent to driving 138 miles in a gas-powered vehicle [Table 1]. The cost of repeated HCV Ab testing was $18,973 (Quest Diagnostics).

**Conclusion:**

We found that repeat HCV Ab testing is common and an opportunity for diagnostic stewardship interventions. Some patients were tested up to 11 times.

The environmental and financial cost of each test depend on insurance, provider, facility infrastructure, and location. Many centers utilize reflex HCV RNA testing ($199.84/test Quest Diagnostics) after a positive HCV Ab test which would have cost $750,799 at our center.

Potential reasons for duplicate orders include gaps in provider access or incomplete review of electronic medical records (EMR), the convenience of order sets, and/or the lack of EMR restrictions. A simple EMR “pop-up” to alert providers when inappropriately ordering repeat testing could be a low-hanging fruit for diagnostic stewardship efforts associated with no harm.

**Disclosures:**

All Authors: No reported disclosures

